# Microglia‐Derived Interleukin‐6 Triggers Astrocyte Apoptosis in the Hippocampus and Mediates Depression‐Like Behavior

**DOI:** 10.1002/advs.202412556

**Published:** 2025-01-30

**Authors:** Shi‐Yu Shen, Ling‐Feng Liang, Tian‐Le Shi, Zu‐Qi Shen, Shu‐Yuan Yin, Jia‐Rui Zhang, Wei Li, Wen‐Li Mi, Yan‐Qing Wang, Yu‐Qiu Zhang, Jin Yu

**Affiliations:** ^1^ Department of Integrative Medicine and Neurobiology School of Basic Medical Sciences Shanghai Medical College Fudan University Shanghai 200032 China; ^2^ State Key Laboratory of Medical Neurobiology and MOE Frontiers Center for Brain Science Department of Translational Neuroscience Jing'an District Centre Hospital of Shanghai Institutes of Brain Science Fudan University Shanghai 200032 China; ^3^ Shanghai Key Laboratory of Acupuncture Mechanism and Acupoint Function Fudan University Shanghai 200433 China

**Keywords:** apoptosis, astrocytes, depression, IL‐6, microglia

## Abstract

In patients with major depressive disorder (MDD) and animal models of depression, key pathological hallmarks include activation of microglia as well as atrophy and loss of astrocytes. Under certain pathological conditions, microglia can inflict damage to neurons and astrocytes. However, the precise mechanisms underlying how activated microglia induced astrocyte atrophy and loss remain enigmatic. In this study, a depression model induced by chronic social defeat stress (CSDS) is utilized. The results show that CSDS induces significant anxiety‐ and depression‐like behaviors, along with notable astrocyte atrophy and apoptosis, microglial activation, and elevated levels of microglial interleukin‐6 (IL‐6). Subsequent studies demonstrate that IL‐6 released from activated microglia promotes astrocyte apoptosis. Furthermore, the knockdown of the P2X7 receptor (P2X7R) in microglia, which is implicated in the stress response, reduces stress‐induced microglial activation, IL‐6 release, and astrocyte apoptosis. Direct inhibition of microglia by minocycline corroborates these effects. The selective knockdown of IL‐6 in microglia and IL‐6 receptors in astrocytes effectively mitigates depression‐like behaviors and reduces astrocyte atrophy. This study identifies microglial IL‐6 as a key factor that contributes to astrocyte apoptosis and depressive symptoms. Consequently, the IL‐6/IL‐6R pathway has emerged as a promising target for the treatment of depression.

## Graphical abstract

In this study, it is observed that chronic social defeat stress (CSDS), an animal model of depression, results in atrophy and apoptosis of hippocampal astrocytes, accompanied by the activation of hippocampal microglia. Furthermore, the deletion and inhibition of microglia, as well as P2X7 receptor (P2X7R) deletion in microglia, mitigate CSDS‐induced astrocyte atrophy and apoptosis and reduce anxiety‐ and depression‐like behaviors. Additionally, CSDS significantly increases the release of IL‐6, and knockdown of IL‐6 in hippocampal microglia and IL‐6R in hippocampal astrocytes significantly enhances astrocyte survival while alleviating CSDS‐induced anxiety‐ and depression‐like behaviors.

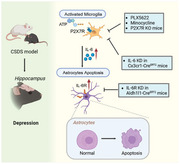

## 1 Introduction

Previous research has suggested that astrocyte atrophy and loss can be either a concurrent manifestation or a contributing factor to depression.^[^ ^1^ ^]^ E.g., individuals suffering from depression often display a reduced density and number of astrocytes in brain regions such as the medial prefrontal cortex (mPFC), hippocampus, and amygdala.^[^ ^2^, ^3^, ^4^ ^]^ Preclinical studies have further indicated that a decline in astrocyte function is linked to the development of depression‐like behaviors.^[^ ^5^, ^6^ ^]^ However, recent studies have shown that activation of A1 reactive astrocytes can lead to depression‐like behaviors in mice.^[^ ^7^, ^8^ ^]^ Thus, astrocytes play a pivotal role in depression; however, the precise mechanisms underlying how stress triggers astrocyte loss and atrophy remain elusive.

The body of evidence supporting the involvement of microglia in the etiology of depression has steadily increased. In numerous preclinical studies, microglial activation and the presence of proinflammatory cytokines have been observed in various brain regions, including the mPFC and hippocampus, and anti‐inflammatory medications appear to alleviate depression symptoms.^[^ ^9^, ^10^, ^11^ ^]^

Microglia are activated upon exposure to pathogen‐associated molecular patterns (PAMPs) and/or endogenous damage‐associated molecular patterns (DAMPs).^[^ ^12^, ^13^ ^]^ Microglia exhibit distinct phenotypes in various pathological contexts in response to cues present in their environment (including different DAMPs or PAMPs). This diverse activation state of microglia is often referred to as disease‐associated microglia (DAM).^[^ ^14^ ^]^ Recent studies indicated that microglia display various activation states and exert different effects on astrocytes in various neurological disorders.^[^ ^15^, ^16^, ^17^ ^]^ In the case of depression, extracellular ATP (eATP) serves as a recognized DAMP responsible for triggering microglial activation through the P2X7 receptor (P2X7R) in response to external stressors (both psychological and physiological stress).^[^ ^18^, ^19^, ^20^, ^21^ ^]^ However, the specific activation state of microglia under conditions of elevated eATP, the factors they release to modulate astrocytes, and the underlying mechanisms driving their chronic activation, leading to astrocyte atrophy and loss of depression, remain poorly understood.

Therefore, our study aimed to investigate pathological alterations in astrocytes and their association with depression‐like behaviors in the context of chronic social defeat stress (CSDS), a well‐established animal model of depression. Furthermore, we sought to identify the molecular mechanisms by which activated microglia mediate stress‐induced astrocyte pathology in vivo and in vitro. The results of this study advance our understanding of the pathogenesis of depression and emphasize the pivotal role of microglia in regulating astrocyte fate in depression.

## 2 Results

### 2.1 Chronic social defeat stress (CSDS) Induces Anxiety‐ and Depression‐Like Behaviors and Astrocyte Atrophy and Apoptosis in the Hippocampus

**Figure 1:**
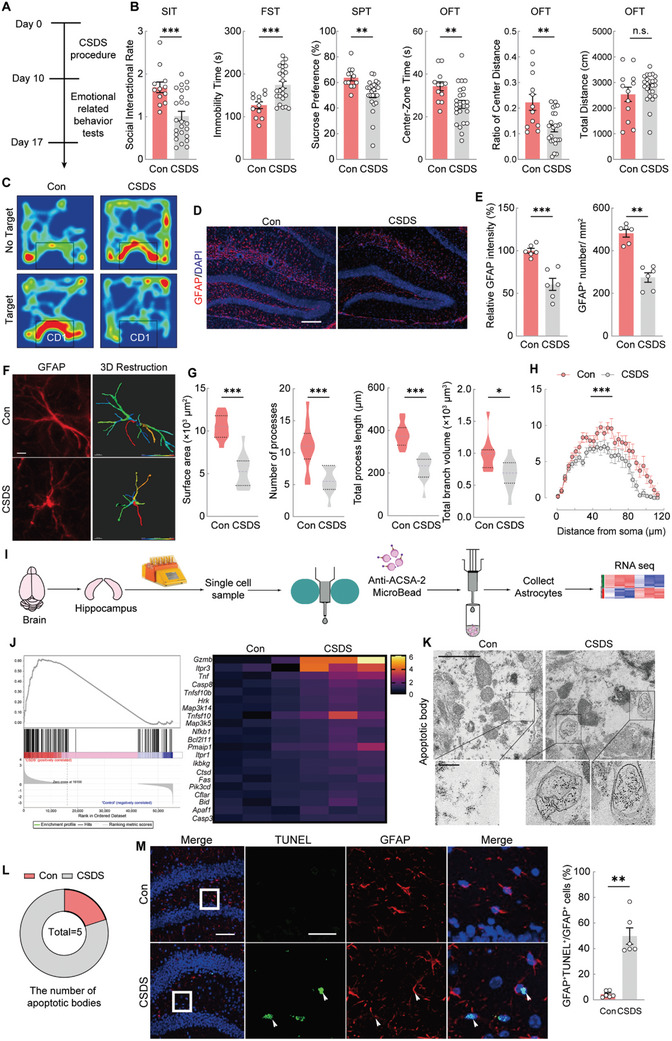
CSDS causes anxiety‐and depression‐ like behaviors as well as hippocampal astrocyte atrophy and apoptosis. A) Experimental timeline of CSDS protocol. B) Performance of Control (Con) and CSDS mice in social interaction test (SIT), forced swimming test (FST), sucrose preference test (SPT), and open field test (OFT). Unpaired *t* test. n_Con_ = 12, n_CSDS_ = 24. C) Representative heat map of trajectories of Con and CSDS mice during SIT test. D) Representative images of GFAP immunostaining (red) in the hippocampus of Con or CSDS mice. Scale bars = 200 µm. E) The immunostaining quantitative analysis of GFAP and number of cells positively labeled for GFAP. Unpaired *t* test. n = 6. F) GFAP fluorescence staining images (left) and 3D morphological reconstruction (right) of astrocytes in the Con and CSDS group. Scale bar = 10 µm. G) Quantitative analyses of the average surface area, the average number of processes, the total branch length, and the total volume of astrocytes. Unpaired *t* test. n = 12. H) Sholl analysis of hippocampal astrocytes in the Con and CSDS group. Two‐way ANOVA. n = 12. I) Schematic diagram of isolating ACSA‐2 positive astrocytes from the hippocampus of control and CSDS mice and followed by RNA‐seq. J) Enrichment plot of GSEA of apoptosis in hippocampal astrocytes of Con and CSDS mice (left), and heat map showing enriched differential expression genes (DEGs) in apoptotic gene set of Con and CSDS group (right). K) Representative TEM images of apoptotic bodies in the hippocampal astrocytes of Con and CSDS mice. Scale bars (overview) = 1 µm and Scale bars (magnified) = 0.2 µm. L) Analysis of the number of apoptotic bodies in the hippocampal astrocytes of Con and CSDS mice. M) Representative images of TUNEL (green), GFAP (astrocytes, red) and a merged image in the hippocampus of Con and CSDS mice, respectively. The percentage of TUNEL^+^, GFAP^+^ double labeled cells in the total GFAP^+^ cells were calculated in a 300 × 300 µm bin in the hippocampal DG (right). Scale bars (overview) = 40 µm and scale bars (magnified) = 20 µm. Unpaired *t* test. n = 6. All data are shown as mean ± S.E. M, * *p* < 0.05, ** *p* < 0.01, *** *p* < 0.001.

CSDS is a well‐established model of depression in C57BL/6J mice.^[^ ^22^, ^23^, ^24^ ^]^ Following the 10‐day CSDS procedure and behavioral testing (**Figure** **1**A), we observed significant depression‐like behaviors, including social avoidance, behavioral despair, and anhedonia, as evidenced by a reduced social interaction ratio (SIR) in the SIT, increased immobility in the FST, and diminished sucrose preference in the SPT. Additionally, anxiety‐like behaviors were marked by less time spent and reduced movement within the central area of the OFT (Figure 1B,C), whereas the overall locomotor activity, measured by the total distance traveled in the OFT, remained unaffected (Figure 1B).

The 10‐day CSDS exposure also caused notable neuropathological changes in astrocytes, consistent with our previous findings in a chronic unpredictable mild stress (CUMS) model.^[^ ^25^ ^]^ These alterations included a significant reduction in GFAP intensity and GFAP^+^ astrocyte count in the hippocampus (Figure 1D,E). Semiautomatic 3D morphometric analysis of astrocytes revealed a diminished surface area, shorter and fewer processes, and reduced branch volume in the CSDS group (Figure 1F,G). Sholl analysis further demonstrated a marked decrease in the complexity of hippocampal astrocyte branching in the CSDS mice (Figure 1H), indicating astrocyte atrophy in response to depressive conditions.

To explore the gene expression changes in astrocytes following CSDS, we performed RNA sequencing on hippocampal astrocytes from control and CSDS‐exposed mice. Single‐cell suspensions of hippocampal tissue were sorted using Anti‐ACSA‐2 microbeads, and RNA was extracted for sequencing (Figure 1I). Results revealed that compared with the control group, the CSDS group exhibited 318 differentially expressed genes (DEGs), of which 280 were upregulated and 38 were downregulated (Figure S1A–D, Supporting Information). Principal component analysis (PCA) indicated low similarity between the groups (Figure S1B, Supporting Information). Gene set enrichment analysis (GSEA) revealed increased expression of apoptosis‐related genes in the hippocampal astrocytes of CSDS mice, including *Gzmb*, *Itpr3*, *Tnf*, and *Caspase8*, potentially contributing to the behavioral changes observed (Figure 1J).

To confirm astrocyte apoptosis post‐CSDS, transmission electron microscopy (TEM) and TUNEL staining were conducted. TEM images revealed apoptotic bodies in the hippocampal astrocytes of CSDS mice, with a notable increase in apoptotic bodies compared to controls (Figure 1K,L). TUNEL staining further demonstrated a significant rise in apoptotic astrocytes, evidenced by more TUNEL^+^ and GFAP^+^ cells in the hippocampus (Figure 1M). These findings indicated that CSDS induces astrocyte atrophy and apoptosis, potentially underlying the neurobiological mechanisms of CSDS‐induced behavioral changes.

### 2.2 Astrocyte Loss Mediates CSDS‐Induced Depression‐Like Behaviors

To investigate whether the inhibition of astrocyte apoptosis could alleviate anxiety‐ and depression‐like behaviors, we treated CSDS mice with the caspase‐3 inhibitor, Z‐DEVD‐FMK (Z‐FMK) (Figure S2A, Supporting Information). Immunofluorescence assays confirmed that Z‐FMK successfully prevented the CSDS‐induced astrocyte loss (Figure S2B, Supporting Information). Interestingly, while Z‐FMK did not affect anxiety‐like behaviors or locomotor activity in CSDS mice, it significantly reversed depression‐like behaviors induced by CSDS (Figure S2C, Supporting Information). These results suggest that astrocyte loss contributes to the development of depression‐like behaviors.

To further explore the role of astrocyte atrophy and loss in depression‐ and anxiety‐like phenotypes induced by CSDS, we used L‐α‐Aminoadipic Acid (L‐AA) to selectively ablate astrocytes. L‐AA disrupts glutamate metabolism and protein synthesis, leading to astrocyte cytotoxicity.^[^ ^26^ ^]^ We assessed its impact on emotional behavior in mice subjected to sub‐CSDS, a model that by itself does not induce depression‐ or anxiety‐like phenotypes (Figures S2D and S3, Supporting Information). The efficiency of L‐AA‐induced astrocyte depletion in the hippocampus was confirmed using immunofluorescence staining (Figure S2E, Supporting Information). L‐AA‐induced astrocyte ablation resulted in pronounced depression‐like behaviors, characterized by a reduced SIR in the SIT, increased immobility in the FST, and diminished sucrose preference in the SPT. Anxiety‐like behaviors were also observed, with less time spent exploring the center zone and a trend toward a reduced center distance ratio in the OFT. However, the locomotor activity remained unaffected by these manipulations (Figure S2F, Supporting Information).

### 2.3 CSDS Induces Microglial Activation and Elevates interleukin‐6(IL‐6) Levels

**Figure 2:**
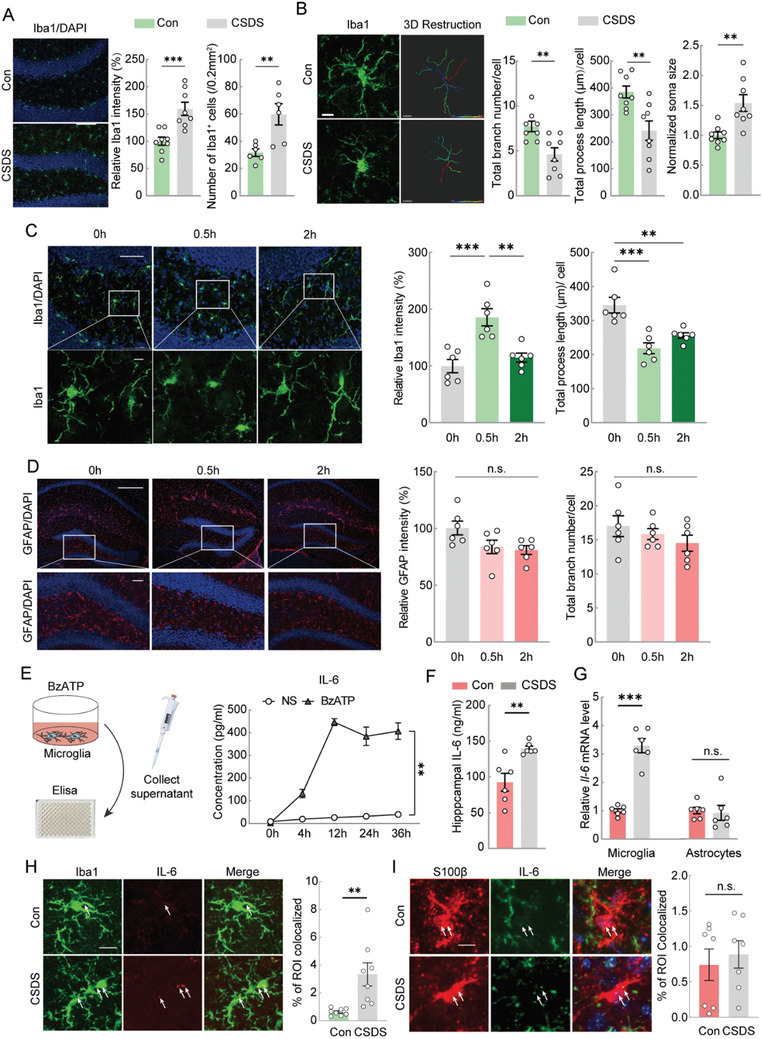
CSDS results in higher levels of microglial IL‐6. A) Representative images (left) of Iba1 immunostaining in hippocampal microglia of mice in the Con group and CSDS group. The histogram (right) showed the relative fluorescence intensity of Iba1(Unpaired *t* test, n = 8) and the number of Iba1^+^ microglia (Unpaired *t* test, n = 6). Scale bar = 100 µm. B) Iba1 fluorescence staining images (left) and 3D morphological reconstruction (middle) in hippocampal microglia of Con and CSDS group. The histogram (right) showed the imaris‐based semi‐automatic quantification of cell morphometry, including total branch number, total process length and normalized soma size of Iba1^+^ microglia. Scale bar = 10 µm. Unpaired *t* test. n = 8. C) Representative images of Iba1 immunostaining in hippocampal microglia at 0 h, 0.5 h, and 2 h after once social defeat stress (left). Overview (top) and magnified (below) are shown. The histogram (right) showed the relative fluorescence intensity and total process length of Iba1^+^ microglia. Scale bars, 50 µm (overview) and 10 µm (magnified). One‐way ANOVA. n = 6. D) Representative images of GFAP immunostaining in hippocampal astrocytes at 0 h, 0.5 h, and 2 h after once social defeat stress. Overview (top) and magnified (below) are shown. The histogram (right) showed the relative fluorescence intensity and total branch number of GFAP^+^ astrocytes. Scale bars, 200 µm (overview) and 50 µm (magnified). One‐way ANOVA. n = 6. E) Drawing of ELISA technique for the detection of inflammatory cytokines secreted by BzATP‐activated cultured microglia (left). The concentration of IL‐6 released by microglia after BzATP treatment for 0 h, 4 h, 12 h, 24 h, and 36 h (right). Two‐way ANOVA. n = 4. F) The concentration of IL‐6 in hippocampus of Con and CSDS group. Unpaired *t* test. n = 6. G) The *Il‐6* mRNA level in hippocampal microglia and hippocampal astrocytes of Con and CSDS group. Unpaired *t* test. n = 6. H) Representative images of Iba1 (microglia, green), IL‐6 (red) and a merged image in the hippocampus of Con and CSDS mice, respectively (left). The histogram (right) showed the colocalization coefficient of Iba1 and IL‐6. Scale bar = 10 µm. Unpaired *t* test. n = 8. I) Representative images of S100β (astrocytes, red), IL‐6 (green) and a merged image in the hippocampus of Con and CSDS mice, respectively (left). The histogram (right) showed the colocalization coefficient of S100β and IL‐6. Scale bar = 10 µm. Unpaired *t* test. n = 6. All data are shown as mean ± S.E. M, * *p* < 0.05, ** *p* < 0.01, *** *p* < 0.001.

Chronic neuroinflammation plays a crucial role in the onset and progression of depression symptoms.^[^ ^11^, ^27^ ^]^ Following 10 days of CSDS, we observed a marked increase in the fluorescence intensity of the microglial marker, Iba1, and an elevated number of microglia (**Figure** **2**A). Detailed 3D morphometric analysis of microglia showed significant increases in the total branch number, process length, and soma size in the hippocampus of CSDS mice (Figure 2B).

Microglia, the brain's primary responders to external stressors, such as inflammation, injury, and psychological stress, are rapidly activated and undergo morphological changes to perform their protective and regulatory functions.^[^ ^28^ ^]^ In C57 mice, the fluorescence intensity of Iba1 in the hippocampus and the length of microglial processes significantly increased by 0.5 h after being attacked by CD1 retired male mice (Figure 2C). In contrast, astrocytes displayed no significant morphological changes, even post 2 h of stress (Figure 2D). Thus, indicating that microglia respond more rapidly to social stress than astrocytes. Furthermore, as dynamic mediators of brain function, microglia influence various processes including astrocyte activation, neural progenitor differentiation, neuronal homeostasis, and synaptogenesis.^[^ ^29^ ^]^ Thus, the initially activated microglia may contribute to the atrophy and loss of astrocytes induced by external stress.

Given that microglia may respond to ATP released by neurons or astrocytes through the P2X7 receptor (P2X7R) in the context of depression,^[^ ^18^, ^20^ ^]^ we treated cultured microglia in vitro with the specific P2X7R agonist BzATP to identify which cytokines are upregulated following P2X7R activation. ELISA results demonstrated that BzATP‐treated microglia exhibited significantly elevated levels of IL‐6 (Figure 2E), without corresponding increases in TNF‐α, IL‐1β, IL‐4, or IL‐10 (Figure S4, Supporting Information). Additionally, there was a substantial increase in the hippocampal IL‐6 levels in the CSDS model (Figure 2F).

As both microglia and astrocytes are sources of IL‐6 in the central nervous system, we further examined the genetic changes in microglia following CSDS. Using anti‐CD11b and anti‐ACSA‐2 microbeads, we sorted the hippocampal microglia and astrocytes, respectively, and subsequently extracted total RNA. RT‐PCR results indicated the upregulation of *Il‐6* mRNA in microglia from the CSDS group, with no significant changes observed in astrocytes (Figure 2G). Furthermore, IL‐6 expression in microglia increased after CSDS (Figure 2H), whereas IL‐6 levels in astrocytes remained unchanged (Figure 2I).

### 2.4 IL‐6 Released by BzATP‐Treated Microglia Prompts Astrocyte Apoptosis

**Figure 3:**
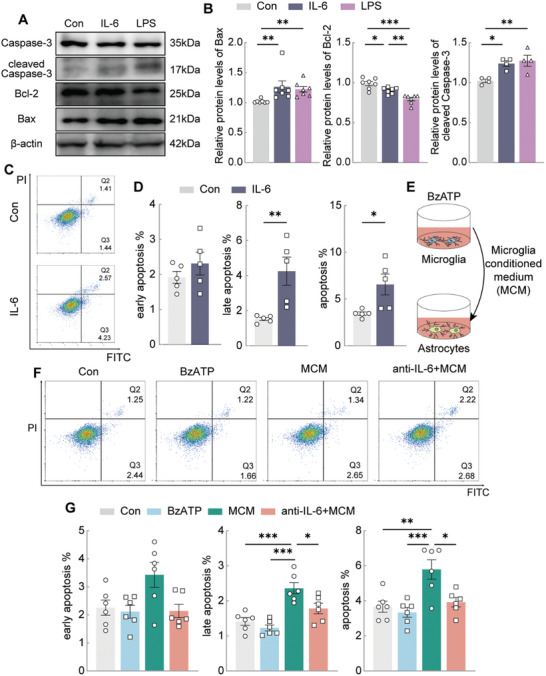
IL‐6 released from activated microglia induces the apoptosis of astrocytes in vitro. A) Representative western blot images of Bax, Bcl‐2 and cleaved Caspase‐3 in astrocytes treated with PBS, IL‐6 and LPS. B) Western blot analysis of Bax (n = 7), Bcl‐2 (n = 7) and cleaved Caspase‐3 (n = 4) expression of astrocytes treated with PBS, IL‐6 and LPS. One‐way ANOVA. C) Representative images of FACS analysis of apoptosis in astrocytes treated with PBS (Con) or IL‐6. D) Apoptosis rate of astrocytes treated with PBS (Con) or IL‐6. Unpaired *t* test. n = 5. E) Drawing of primary cultured astrocytes stimulated with BzATP‐treated microglia conditioned medium (MCM). F) Representative images of FACS analysis of astrocytes treated with PBS (Control), BzATP, MCM alone or MCM combined with IL‐6 antibody. G) Apoptosis rate of astrocytes treated with Con, BzATP, MCM alone or MCM combined with IL‐6 antibody. One‐way ANOVA. n = 6. All data are shown as mean ± S.E.M, * *p* < 0.05, ** *p* < 0.01, *** *p* < 0.001.

To further explore whether IL‐6 directly induces astrocyte apoptosis, we treated astrocytes with IL‐6 at the same concentration as that released by BzATP‐activated microglia (500 pg mL⁻^1^). We also examined apoptosis‐related protein expression using western blotting. After 24 h of IL‐6 treatment, we observed an increase in Bax (a pro‐apoptotic protein) and cleaved Caspase‐3 (an apoptosis marker) and a decrease in Bcl‐2 (an anti‐apoptotic protein) (**Figure** **3**A,B). Additionally, Annexin V‐FITC/PI flow cytometry revealed late‐stage and total apoptosis in astrocytes after 24 h of IL‐6 exposure (Figure 3C,D).

To confirm whether IL‐6 is the primary factor mediating microglia‐induced astrocyte apoptosis, we activated microglia in vitro with the specific P2X7R agonist BzATP. We then exposed cultured astrocytes to microglia‐conditioned media (MCM) to assess the effect of P2×7R‐activated microglia on astrocyte apoptosis (Figure 3E). Subsequently, flow cytometry showed that MCM induced astrocyte apoptosis, whereas BzATP alone did not affect the early, late, or total apoptosis levels (Figure 3F,G). Furthermore, pretreatment with IL‐6 antibody 6 h before MCM exposure significantly reduced astrocyte apoptosis (Figure 3F,G). These findings suggest that inflammation driven by P2×7R‐activated microglia promotes astrocyte apoptosis and that IL‐6 plays a crucial role in this process.

### 2.5 Microglia Contribute to CSDS‐Induced Astrocyte Atrophy

Based on these results, we hypothesized that stress‐induced microglial activation contributes to astrocyte atrophy and hippocampal loss. To test this hypothesis, we systemically administered PLX5622, a CSF1R inhibitor (Figure S5A,B, Supporting Information), which resulted in a 90% depletion of hippocampal microglia (Figure S5C, Supporting Information). Behavioral tests showed that PLX5622 prevented depression‐ and anxiety‐like behaviors induced by CSDS (Figure S5D, Supporting Information) and mitigated astrocyte atrophy and loss associated with CSDS (Figure S5E, Supporting Information).

**Figure 4:**
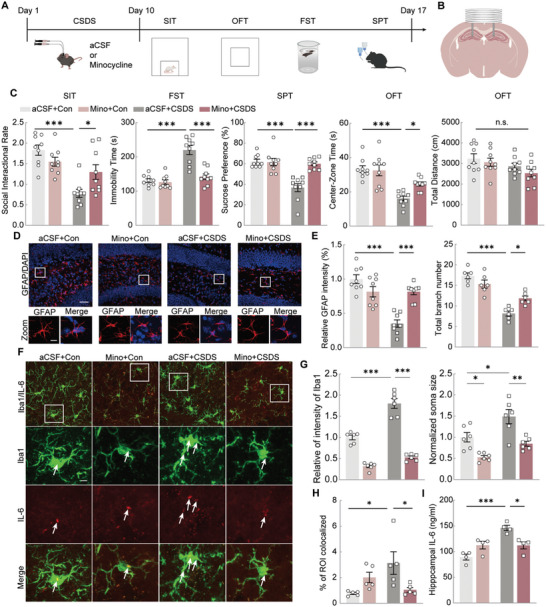
Minocycline alleviates anxiety and depression‐like behaviors and decreases astrocytes atrophy. A) Experimental timeline of Minocycline (Mino) treatment, CSDS protocol and behavioral tests. B) Drawing of the cannula implanted sites. C) Performance of Con and CSDS mice treated with Mino or aCSF in SIT, FST, SPT, and OFT. Two‐way ANOVA. n = 9. D) Representative images of GFAP (red) staining in the hippocampus of Con and CSDS mice treated with Mino or aCSF via an infusion cannula. Scale bars (overview) = 40 µm and scale bars (magnified) = 10 µm. E) Quantitative immunostaining and total branch numbers analysis of GFAP^+^ cells in Con and CSDS mice treated with Mino or aCSF via an infusion cannula. Two‐way ANOVA. n = 8. F) Representative images of Iba1 (green) and IL‐6 (red) staining in the hippocampus of Con and CSDS mice treated with Mino or aCSF via an infusion cannula. Scale bars (overview) = 40 µm and scale bars (magnified) = 10 µm. G) Quantitative immunostaining and normalized soma size analysis of Iba1^+^ cells in Con and CSDS mice treated with Mino or aCSF via an infusion cannula. Two‐way ANOVA. n = 6. H) The histogram showed the colocalization coefficient of Iba1 and IL‐6 in Con and CSDS mice treated with Mino or aCSF. Two‐way ANOVA. n = 5. I) The concentration of IL‐6 in hippocampus in Con and CSDS mice treated with Mino or aCSF. Two‐way ANOVA. n = 4. All data are shown as mean ± S.E.M, * *p* < 0.05, ** *p* < 0.01, *** *p* < 0.001.

However, because the systemic administration of PLX5622 also affects macrophages throughout the body, we used minocycline (Mino), a microglial inhibitor, delivered directly into the hippocampus via an implanted cannula (**Figure** **4**A,B). As anticipated, hippocampal microinjection of Mino inhibited microglial activity (Figure 4A,B), prevented depression‐ and anxiety‐like behaviors caused by CSDS, and did not affect locomotor activity (Figure 4C). In addition, Mino also significantly reversed astrocyte atrophy and loss (Figure 4D,E). These results suggest that hippocampal microglial activation triggered by CSDS plays a critical role in astrocyte survival and the development of depressive symptoms.

We assessed Mino's effects on microglial morphology and IL‐6 expression. Mino significantly reduced the CSDS‐induced increase in microglial fluorescence intensity and soma size (Figure 4E–G). Additionally, Mino treatment lowered the elevated expression of IL‐6 in the microglia (Figure 4E,H) and decreased the overall IL‐6 concentration in the hippocampus (Figure 4I).

Several studies have demonstrated that activation of P2X7R, which is specifically expressed in microglia, mediates microglial responses to eATP and contributes to anxiety‐ and depression‐like behaviors.^[^ ^20^, ^30^, ^31^ ^]^ However, whether P2X7R activation is involved in microglia‐driven astrocyte atrophy and apoptosis remains unclear. To explore this, we investigated whether P2X7R depletion could alleviate CSDS‐induced astrocyte atrophy, loss, and anxiety‐ and depression‐like behaviors.

**Figure 5:**
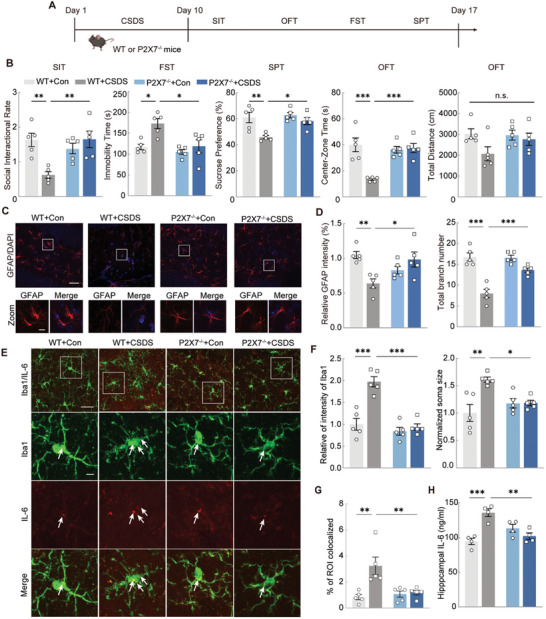
P2X7R deletion alleviates anxiety and depression‐like behaviors and decreases astrocytes atrophy. A) Experimental timeline of WT and P2X7R^−/−^ mice treated with CSDS protocol and behavioral tests. B) Performance of WT and P2X7R^−/‐^ mice treated with Con or CSDS in SIT, FST, SPT, and OFT. Two‐way ANOVA. n = 5. C) Representative images of GFAP (red) staining in the hippocampus of WT and P2X7R^−/−^ mice treated with Con or CSDS. Scale bars (overview) = 40 µm and scale bars (magnified) = 10 µm. D) Quantitative immunostaining and total branch numbers analysis of GFAP^+^ cells in WT and P2X7R^−/−^ mice treated with Con or CSDS. Two‐way ANOVA. n = 5. E) Representative images of Iba1 (green) and IL‐6 (red) staining in the hippocampus of WT and P2X7R^−/−^ mice treated with Con or CSDS. Scale bars (overview) = 40 µm and scale bars (magnified) = 10 µm. F) Quantitative immunostaining and normalized soma size analysis of Iba1^+^ cells in WT and P2X7R^−/−^ mice treated with Con or CSDS. Two‐way ANOVA. n = 5. G) The histogram showed the colocalization coefficient of Iba1 and IL‐6 in WT and P2X7R^−/−^ mice treated with Con or CSDS. Two‐way ANOVA. n = 5. H) The concentration of IL‐6 in hippocampus in WT and P2X7R^−/−^ mice treated with Con or CSDS. Two‐way ANOVA. n = 4. All data are shown as mean ± S.E.M, * *p* < 0.05, ** *p* < 0.01, *** *p* < 0.001.

For this purpose, we used P2X7R knockout (P2X7R^−/−^) mice in a 10‐day CSDS model, following established behavioral testing protocols (**Figure** **5**A). Notably, P2X7R deletion prevented the development of depression‐ and anxiety‐like behaviors induced by CSDS, without affecting locomotor activity (Figure 5B). Furthermore, P2X7R knockout mice exhibited reduced astrocyte atrophy and loss associated with CSDS (Figure 5C,D).

In addition, P2X7R deletion significantly decreased the elevated microglial fluorescence intensity and enlarged soma size observed in CSDS‐treated mice (Figure 5E,F). It also attenuated the CSDS‐induced increase in IL‐6 expression in the microglia (Figure 5E,G) and lowered hippocampal IL‐6 concentrations (Figure 5H).

### 2.6 IL‐6 Mediates Astrocyte Atrophy in the Hippocampus and CSDS‐Induced Depression‐Like Behaviors

Building on the finding that activated microglia release IL‐6, leading to astrocyte apoptosis in vitro, we further investigated whether IL‐6 released by microglia mediates astrocyte atrophy and apoptosis and contributes to anxiety‐ and depression‐like behaviors in vivo.

**Figure 6:**
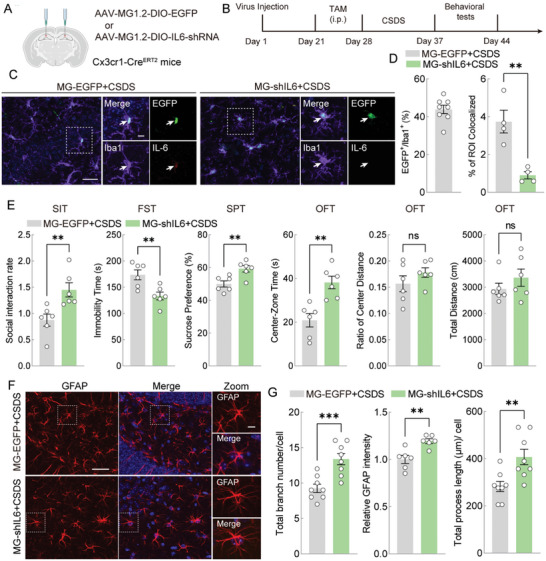
Knockdown of microglial IL‐6 alleviates CSDS‐induced depressive‐like phenotype as well as hippocampal astrocytes atrophy. A) Schematic diagram showing the experimental strategy for IL‐6 knockdown of hippocampal microglia in vivo. B) Timeline of experimental strategy for IL‐6R knockdown of hippocampal astrocytes in vivo. C) Representative images of the AAV‐infected microglia (EGFP), Iba1 (cy5), IL‐6 (red) in hippocampus. Scale bars (magnified, left) = 10 µm and scale bars (overview, right) = 40 µm. D) The proportion of EGFP^+^ cells in the number of Iba1^+^ microglia is shown left. n = 8. And the histogram (right) showed the colocalization coefficient of Iba1 and IL‐6. Unpaired *t* test. n = 4. E) Performance of Cx3cr1‐Cre^ERT2^ mice treated with AAV‐DIO‐EGFP and AAV‐DIO‐shil6 after CSDS in SIT, FST, SPT, and OFT. Unpaired *t* test. n = 6. F) Representative images of GFAP (red) staining in the hippocampus of CSDS mice treated with AAV‐DIO‐EGFP and AAV‐DIO‐Shil6r. Scale bars (overview) = 40 µm and scale bars (magnified) = 10 µm. G) Imaris‐based semi‐automatic quantification of total branch numbers (n = 7), relative GFAP intensity (n = 6) and total branch length analysis (n = 8) of GFAP^+^ astrocytes. Unpaired *t* test. All data are shown as mean ± S.E.M, * *p* < 0.05, ** *p* < 0.01, *** *p* < 0.001.

To specifically knock down IL‐6 in hippocampal microglia, we used a novel viral vector capable of specifically targeting microglia.^[^ ^32^ ^]^ This vector was injected into the bilateral hippocampal regions of Cx3cr1‐Cre^ERT2^ mice (**Figure** **6**A). After three weeks of viral expression, the mice were administered Tamoxifen (i.p.) for seven days to induce Cre enzyme expression, followed by CSDS modeling and behavioral testing (Figure 6B). This method resulted in successful infection of ≈42% of the hippocampal microglia (Figure 6C,D).

Remarkably, IL‐6 knockdown in hippocampal microglia significantly reduced social avoidance, depression‐like behaviors, and anxiety‐like behaviors induced by CSDS without affecting overall motor activity (Figure 6E). Additionally, IL‐6 knockdown effectively reversed CSDS‐induced astrocyte atrophy, as evidenced by increased GFAP intensity and enhanced number and length of astrocyte branches (Figure 6F,G). These results suggest that microglial IL‐6 plays a key role in mediating astrocyte atrophy and mood‐related behaviors following CSDS.

**Figure 7:**
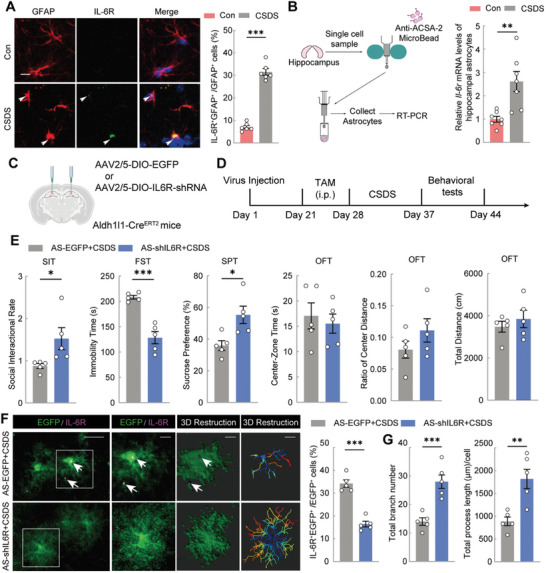
Knockdown of astrocytes IL‐6 receptor (IL‐6R) alleviates CSDS‐induced depressive‐like phenotype as well as hippocampal astrocytes atrophy. A) Representative images of GFAP (red) and IL‐6R (green) staining in the hippocampus of Con and CSDS mice. The proportion of IL‐6R^+^ / GFAP^+^ cells in the number of GFAP^+^ cells is shown (right). Scale bars = 25 µm. Unpaired *t* test. n = 6. B) Schematic diagram of isolating ACSA‐2 positive astrocytes from the hippocampus of Con and CSDS mice followed by RT‐PCR (left). Hippocampal astrocyte expression level of *Il‐6r* mRNA in Con and CSDS group (right). Unpaired *t* test. n = 6. C) Schematic diagram showing the experimental strategy for IL‐6R knockdown of hippocampal astrocytes in vivo. D) Timeline of experimental strategy for IL‐6R knockdown of hippocampal astrocytes in vivo. E) Performance of Aldh1l1‐Cre^ERT2^ mice treated with AAV‐DIO‐EGFP and AAV‐DIO‐shIL‐6R of after CSDS in SIT, FST, SPT, and OFT. Unpaired *t* test. n = 5. F) Representative images of the AAV‐infected astrocytes in mouse hippocampus (left). Scale bars (magnified, left) = 10 µm and scale bars (overview, right) = 40 µm. The proportion of IL‐6R^+^ / EGFP^+^ cells in the number of EGFP^+^ astrocytes is shown right. Unpaired *t* test. n = 5. G) Imaris‐based semi‐automatic quantification of total branch numbers and total branch length analysis of EGFP^+^ astrocytes. Unpaired *t* test. n = 5. All data are shown as mean ± S.E.M, * *p* < 0.05, ** *p* < 0.01, *** *p* < 0.001.

Subsequently, we examined IL‐6R expression in the hippocampal astrocytes and found a significant increase in the number of IL‐6R‐expressing astrocytes after CSDS (**Figure** **7**A). In parallel, we assessed *Il‐6r* mRNA levels in astrocytes isolated by magnetic bead sorting, which revealed a significant upregulation of *Il‐6r* in astrocytes following CSDS (Figure 7B).

Based on these results, we knocked down IL‐6R in hippocampal astrocytes of Aldh1l1‐Cre^ERT2^ mice and assessed the changes in CSDS‐induced behavioral phenotypes and astrocyte loss (Figure 7C,D). The results showed that IL‐6R knockdown in hippocampal astrocytes mitigated social avoidance and depression‐like behaviors in CSDS mice without affecting locomotor activity. However, anxiety‐like behaviors remained unchanged (Figure 7E). Additionally, IL‐6R knockdown successfully reduced IL‐6R expression in astrocytes (Figure 7F) and reversed CSDS‐induced astrocyte atrophy, as evidenced by the increased branch.

Given that numerous studies suggest the IL‐6 trans‐signaling pathway may contribute to depression,^[^ ^33^ ^]^ we treated the CSDS mice with sgp130, an inhibitor of this pathway (Figure S6A, Supporting Information). The behavioral results indicated that sgp130 significantly alleviated the anxiety‐ and depression‐like behaviors (Figure S6B–D, Supporting Information) and improved the atrophy of astrocytes induced by CSDS (Figure S6E, Supporting Information).

Overall, these findings indicate that knocking down IL‐6 in hippocampal microglia and IL‐6R in hippocampal astrocytes, along with inhibition of the IL‐6 trans‐signaling pathway using sgp130, significantly alleviates depression‐like behaviors and reduces astrocyte atrophy in CSDS mice.

## 3 Discussion

In this study, we observed that CSDS, an animal model of depression, caused apoptosis and atrophy in hippocampal astrocytes as well as the activation of hippocampal microglia. Moreover, CSDS significantly altered the morphology and function of the microglia, thereby promoting the release of IL‐6. Furthermore, the deletion and inhibition of microglia, along with P2X7R deletion, mitigated CSDS‐induced astrocyte atrophy and anxiety‐ and depression‐like behaviors. In vitro experiments have demonstrated that IL‐6 released by BzATP‐activated microglia leads to astrocyte apoptosis. Notably, the knockdown of IL‐6 in hippocampal microglia and IL‐6R in hippocampal astrocytes significantly improved astrocyte survival and alleviated anxiety‐ and depression‐like behaviors induced by CSDS.

Postmortem findings in most patients with depression suggest that astrocyte atrophy or loss may represent an important pathological phenotype that is closely associated with major depression.^[^ ^2^, ^3^, ^4^, ^34^ ^]^ However, there is an ongoing debate regarding the pathological changes in astrocytes and their contribution to depression. It is widely accepted that loss of astrocyte function results in depression‐like behaviors.^[^ ^5^, ^6^, ^35^ ^]^ Our study provides additional insight into the role of astrocytes in the pathogenesis of depression. Our data demonstrate that CSDS leads to significant atrophy and apoptosis of astrocytes in the hippocampus, and that inhibition of astrocyte apoptosis in CSDS mice notably alleviates depression‐like behavior. Consistent with the findings of Banasr et al.^[^ ^26^ ^]^ our results suggest that hippocampal astrocyte atrophy and apoptosis represent the central neurobiological mechanisms underlying depression.

Liddelow SA et al. demonstrated that A1 reactive astrocytes are induced by classically‐activated neuroinflammatory microglia through the secretion of IL‐1α, TNF‐α, and C1q, a phenomenon observed in human neurodegenerative diseases such as Alzheimer's, Huntington's, Parkinson's, amyotrophic lateral sclerosis, and multiple sclerosis.^[^ ^16^ ^]^ While some studies have suggested A1 reactive astrocytes contribute to depression‐like behaviors and memory deficits observed in mice,^[^ ^8^ ^]^ Liu et al. introduced a novel drug, UNC9995, which improved depression‐like behaviors by preventing inflammation‐induced astrocyte loss in CSDS and CUMS mice.^[^ ^36^ ^]^

Furthermore, microglia exhibit differential activation and can influence astrocyte fate under various pathological conditions, such as spinal cord injury and traumatic brain injury.^[^ ^15^, ^17^ ^]^ Collectively, our findings suggest that in depression, proinflammatory‐activated microglia are responsible for the atrophy and apoptosis of astrocytes, while in different pathological contexts, microglia play diverse roles in determining the fate of astrocytes.

As previously mentioned, various diseases elicit differential microglial activation that determines the fate of astrocytes, leading to either A1 (neurotoxic) outcomes^[^ ^16^ ^]^ or A2 (neuroprotective) responses^[^ ^17^ ^]^ mediated by cytokines such as the TNF‐α/IL‐1α/C1q cytokines cocktail^[^ ^16^ ^]^ or the IL‐1β/TNF‐α/IL‐6 cytokines cocktail.^[^ ^17^ ^]^ However, the activation of P2X7R, which mimics the high eATP levels induced by chronic stress,^[^ ^18^ ^]^ notably leads to a significant increase in microglial‐released IL‐6, as indicated by our findings. These results align with the observations of Liu et al.,^[^ ^36^ ^]^ who demonstrated significant upregulation of IL‐6 in the hippocampus of susceptible mice subjected to CSDS and CUMS.

However, the cellular and molecular mechanisms by which elevated IL‐6 levels contribute to the progression of depression remain poorly understood. IL‐6 influences the risk of depression through two distinct signaling pathways: classical IL‐6 signaling and trans IL‐6 signaling. In classical IL‐6 signaling, IL‐6 binds to membrane‐bound receptors (mIL‐6R), regulating microglial activity, contributing to immune regulation, and modulating various cytokines.^[^ ^37^, ^38^, ^39^ ^]^ In trans IL‐6 signaling, IL‐6 binds to the soluble IL‐6 receptor (sIL‐6R) in circulation, forming an IL‐6/sIL‐6R complex capable of activating cells lacking mIL‐6R. Trans IL‐6 signaling is generally considered more potent than classical IL‐6 signaling and plays a significant role in neuroinflammation and behavior.^[^ ^40^ ^]^

Glycoprotein 130 (gp130) is a key transmembrane protein required by IL‐6 receptor family members to engage in trans‐signaling, which triggers a pro‐inflammatory response.^[^ ^33^ ^]^ Studies have indicated that sgp130Fc, which selectively binds to the IL‐6/sIL‐6R complex, significantly lowers IL‐6 levels in treated animals compared to placebo.^[^ ^41^ ^]^ In our studies, we find that sgp130Fc treatment in hippocampus effectively alleviates the depression and anxiety behaviors induced by CSDS, and also alleviates atrocytes atrophy. These findings suggest that sgp130Fc, a specific inhibitor of trans IL‐6 signaling, may hold promise as a therapeutic agent for depression.

In addition to astrocytes, IL‐6 may contribute to the pathological process of depression by influencing neural function and neurogenesis. Increasing evidence suggests that IL‐6 plays a significant role in neuronal development, survival, functional regulation, and neurogenesis under both normal and pathological conditions, including depression models.^[^ ^42^, ^43^ ^]^ Additionally, IL‐6 inhibited adult neurogenesis in the DG region.^[^ ^44^ ^]^

Numerous clinical studies have confirmed the association between IL‐6 and depression.^[^ ^45^ ^]^ Meta‐analyses have demonstrated that serum/plasma IL‐6 levels are elevated in patients with major depressive disorder (MDD) compared to those in control groups.^[^ ^46^, ^47^, ^48^ ^]^ However, serum IL‐6 levels may be influenced by multiple factors, such as gender, and may not directly reflect the severity of depression.^[^ ^49^ ^]^ Conversely, other studies have indicated that elevated central IL‐6 concentrations may contribute to the onset of depression.^[^ ^50^ ^]^ Moreover, inhibition of IL‐6 signaling is regarded as a viable strategy for treating MDD.^[^ ^51^ ^]^ These findings suggest that IL‐6 plays an indispensable role in the pathogenesis of depression.

However, this study has several limitations. For instance, we did not include female animals in the CSDS model. This limitation arises because the CSDS model is based on the territorial behavior of male rodents, restricting its reliable application to male C57BL/6 mice.^[^ ^52^ ^]^ Consequently, we were unable to include female C57BL/6 mice in this study, owing to the specific nature of the model. Nevertheless, several studies have investigated sex differences in other animal models of depression, demonstrating that behavioral phenotypes can vary between male and female mice under identical experimental conditions. Moreover, the microglia in male and female mice exhibited different responses to stimulation.^[^ ^53^, ^54^, ^55^, ^56^, ^57^, ^58^, ^59^ ^]^ Based on these findings, we believe that further research is necessary to explore potential sex differences.

Overall, our study sheds light on the pathophysiology of depression from the perspective of glial cell crosstalk and potential intracellular mechanisms involved in astrocyte atrophy and apoptosis. These findings provide new perspectives on the pathogenesis of depression.

## 4 Conclusion

In conclusion, the present study demonstrated that profound pathological alterations manifest in both astrocytes and microglia throughout the progression of depression, with microglial activation contributing to astrocyte atrophy and apoptosis. Furthermore, IL‐6 released from activated microglia plays a crucial role in the development of depression by inducing astrocyte atrophy and apoptosis. In addition, the IL‐6/IL‐6R pathway represents a potential target for depression treatment, offering valuable insights into depression therapy.

## 5 Experimental Section

### Animals

Male C57BL/6 J mice (7–9 weeks, Shanghai SLAC Laboratory Animal Co. Ltd.) and male retired CD1 mice (8–9 months, Vital River Laboratories, Beijing, China) were housed in a conditioned environment with 12 h light/dark cycle, 22–24 °C, and 55 ± 5% humidity. Before experiments began, all experimental mice were allowed to adjust to the environment for at least 1 week and were fed ad libitum. P2X7R^−/−^ mice (JAX stock no.005576) and Cx3cr1‐ Cre^ERT2^ mice (JAX stock no.021160) were obtained from Jackson Lab, and Aldh1l1‐Cre^ERT2^ mice were donated by Prof. Tian‐Ming Gao.^[^ ^60^ ^]^ All protocols were approved by the Experimental Animal Ethics Committee of Shanghai Medical College, Fudan University.

### Primary Cell Culture—Primary Astrocytes Culture

Primary astrocytes were dissociated from 1‐3‐day‐old newborn C57 mice, the cortex and hippocampus were collected, and meninges and basal ganglia were removed from the surface of cortex and hippocampus carefully. Then the cleaned tissue was blown up into single cells using a Pasteur dropper, and after centrifugation, cells were resuspended in completed medium (Dulbecco's modified Eagle's medium (DMEM) (Corning, USA) supplemented with 10% fetal bovine serum (FBS) (Hyclone) and 1% penicillin/streptomycin). The culture‐completed medium was changed with fresh completed medium 24 h later and then changed every 3 days. Cells were cultured in a humidified incubator at 37 °C and 5% CO_2_.

### Primary Cell Culture—Isolation and Purification of Primary Microglia Culture

In this study, microglia were isolated and purified by mild enzymatic method.^[^ ^61^ ^]^ Mild trypsin solution was prepared by mixing 0.25% trypsin solution with DMEM/F12 at a ratio of 1:3. Incubation of the unpurified mixed glial cells with mild trypsin for 15 min at 37 °C resulted in the detachment of the entire layer of the upper layer of the astrocytes, after which many cells remained attached to the bottom of the plate. The remaining cells at the bottom were microglia. Microglia were collected by cell scrapers and then split onto 24‐well culture plates at 1.5 × 10^5^/mL.

### Social Defeat Stress Models—Chronic Social Defeat Stress (CSDS) Model

The CSDS paradigm was conducted as previously described.^[^ ^24^, ^52^ ^]^ In brief, the C57BL/6J mice were exposed to a CD1 aggressor with reliable attack latencies for 10 min every day, and the CD1 mice were novel for each C57 mouse for 10 consecutive days. After social defeat procedure, the defeated mice were placed on the other side to the aggressive CD1 and exposed to visual, olfactory, and auditory stimulation from CD1 for 24 h, until next physical defeat occurred. Control mice were housed separately in pairs in one cage and switched between 10 different C57BL/6 J mice for 10 consecutive days.

### Social Defeat Stress Models—Sub‐Threshold Social Defeat Stress (Sub‐CSDS) Model

Mice were subjected to a sub‐threshold social defeat stress as described previously.^[^ ^62^, ^63^ ^]^ C57 mice were exposed to a novel CD1 mouse for 5 min and then separated from CD1 for 15 min to receive the visual, olfactory, and auditory stimulation from CD1. After repeating this program for 3 times, the defeated mice were housed in their individual cages. 24 h after the last session, animals were subjected to behavioral tests.

### Behavioral Tests—Social Interaction Test (SIT)

SIT was conducted to assess the social behavior of C57BL/6 J mice.^[^ ^52^ ^]^ The first 2.5 min session was “no target” session, each C57BL/6 J mouse was permitted free exploration of the arena (50 cm length × 50 cm width × 42 cm height) with an empty cage (10 cm length × 10 cm width × 42 cm height), which allowed for visual, olfactory, and auditory contact. During the second 2.5 min session (“target” session), the empty cage was replaced by a cage with an unfamiliar aggressive CD1 mouse. The range of 10 cm over the cage is defined as interaction zone, and time spent in the interaction zone by each C57BL/6 J mouse was measured. Furthermore, the SIR was defined as the time spent in the interaction zone with a target (CD1 mouse), to the time spent in the interaction zone with no target.

### Behavioral Tests—Open Field Test (OFT)

OFT was conducted to evaluate the anxiety‐ like behavior. The apparatus consisted of a square arena (50 cm × 50 cm × 42 cm height), and during the test, mice were gently placed in the center of the square and were allowed to explore the arena for 5 min. The test took place in dim light (15 lux) and after each test, 75% alcohol was used to remove the residual odor.

### Behavioral Tests—Forced Swimming Test (FST)

FST is widely used to assess the depression‐like behavior of mice. During this test, each mouse was gently placed in a glass cylinder (16 cm in diameter and 32 cm in height) containing 20 cm of water (25 ± 1 °C) for 5 min, and the immobility time was recorded. In this test, the increase in immobility time implies an increase in depression‐like behavior.

### Behavioral Tests—Sucrose Preference Test (SPT)

Before the formal testing, mice were housed individually and habituated with 2 identical bottles of 2% sucrose and water for 24 h, in order to prevent possible side preference in the test, the positions of the bottles were exchanged for 12 h. Then the experimental mice were water‐ and food‐ deprived for 24 h. Mice were tested to drink from two bottles for 8 h, one bottle contained sucrose solution and the other contained water, and the positions of the bottles were exchanged after 4 h for the previous reason. The consumption of water, sucrose solution, and total intake of liquids were assessed in all groups. The preference for sucrose was determined by quantifying the ratio between the sucrose solution consumed and the overall liquid intake.

### Pharmacological Intervention—In Vivo Pharmacological Administration

L‐α‐aminoadipic acid (L‐AA) (Cat. No. C5474, APExBIO) is a glutamine synthetase inhibitor. It was dissolved in aCSF to 200 μм, and 0.5 µL of both sides of the vehicle or L‐AA was delivered in hippocampus by implanted guide cannula (RWD) continuously until sacrificed.^[^ ^64^ ^]^

Z‐DEVD‐FMK (Z‐FMK) (Cat. No. S7312, Selleck) was dissolved in DMSO and then dissolved in aCSF at the final concentration of 160 ng µL⁻^1^, 0.5 µL⁻^1^ of both sides of vehicle or Z‐FMK was injected in hippocampus at 30 min before social defeat, and vehicle or Z‐FMK was delivered in hippocampus through cannula continuously during CSDS paradigm and behavioral tests until sacrificed.^[^ ^65^ ^]^

Minocycline (Mino) (Cat. No. HY‐17412A, MCE) was dissolved in aCSF to 40 nм, and 250 nL/side was and the vehicle solution (aCSF, 250 nL/side) was locally applied as a control. aCSF or Mino was delivered in hippocampus through cannula continuously during CSDS paradigm and behavioral tests until sacrificed.^[^ ^66^ ^]^

PLX5622 (PLX) (Cat. No. HY‐16749, MCE) was to pharmacologically ablate brain microglia. It was mixed in AIN‐76A rodent chow (290 mg PLX per kilogram of diet, Plexxikon), and provided to the animals ad libitum. PLX was fed continuously during CSDS paradigm and behavioral tests until sacrificed.^[^ ^66^ ^]^

Tamoxifen (TAM) (Cat. No. T5648, Sigma‐Aldrich) was dissolved in 100% ethanol (20 mg mL⁻^1^) as a stock solution and suspended in corn oil (Cat. No. C8267, Sigma‐Aldrich) to make a final concentration of 20 mg mL⁻^1^. To activate the Cre activity of Cx3cr1‐Cre^ERT2^ and Aldh1l1‐Cre^ERT2^ after virus injection, TAM was injected intraperitoneally at 100 mg k^−1^g for 7 days to induce Cre recombinase expression.^[^ ^66^ ^]^

Recombinant Mouse gp130 Fc Chimera (sgp130fc) (Catalog #: 468‐MG, R&D Systems) was reconstituted at 100 µg mL⁻^1^ in sterile PBS. For cannulated (internal diameter 0.34 mm, RWD) mice, 0.5 µL (0.5 µL/min) of both sides of sgp130fc or sterile PBS were injected in the hippocampus, and the injection cannula was maintained in place for 1min at the conclusion of infusion. Sgp130fc or sterile PBS was administered on day 1 and day 5 of CSDS paradigm.^[^ ^67^ ^]^

### Pharmacological Intervention—In Vitro Incubations with Compounds to Interfere with Astrocytes and Microglia

BzATP triethylammonium salt (BzATP) (Cat. No. HY‐136254, MCE) was used to stimulate P2X7R in microglia and then activated microglia. BzATP primed‐primary microglia were treated with 100 μм for 24 h and then the supernatant was collected as conditioned media from BzATP‐treated microglia (MCM).^[^ ^68^ ^]^ MCM was then transferred to primary astrocytes for another 24 h for detecting the crosstalk between astrocytes and microglia.

Recombinant Mouse IL‐6 Protein (IL‐6) (Cat. No. 406‐ML, R&D) was applied to primary astrocytes at a concentration of 500 pg mL⁻^1^ for different time points (0 h, 6 h, 12 h, and 24 h).

Mouse IL‐6 antibody (Cat. No. MAB406, R&D) was applied to primary astrocytes at a concentration of 100 ng mL⁻^1^ for 6 h and then stimulated with MCM for 24 h.

### Isolating of Hippocampal Astrocytes and Hippocampal Microglia

Bilateral hippocampus were extracted after the mice were sacrificed, and Adult Brain Dissociation Kit (Miltenyi Biotec) was used to isolate hippocampus into individual cells with cellular activity. Briefly, PBS containing 10% FBS was added to resuspend the cells, then FcR Blocking Reagent was added, then Anti‐ACSA‐2 magnetic beads for astrocytes (or Anti‐CD11b magnetic beads for microglia) were added and the cells were treated at 4 °C in the dark for 15min. Then the cells were centrifuged to collect the precipitate, which was then resuspended in PBS containing 10% FBS. After that, the cells were added to the LS column. In this step, the cells combined with the magnetic beads would remain in the LS column, while the unbound cells were washed away. Finally, the LS column was quickly rinsed with PBS containing 10% FBS, and the cells were collected. After removing the supernatant, the sorted astrocytes and microglia were obtained.

### RT‐PCR Analysis

The hippocampal astrocytes or hippocampal microglia were homogenized in Trizol reagent (Takara, Kusatsu, Shiga, Japan) for total RNA extraction. The purity and concentration of the samples were assessed by the Nanodrop Spectrophotometer (NanoDrop ND‐2000; NanoDrop Technologies, Wilmington, USA). The adoptable ratios of the absorbances at wavelengths of 260 nm and 280 nm were 1.8–2.0, and the unqualified samples were eliminated. Subsequently, ≈1 µg of RNA was used for RT‐PCR assay, performed using TB green Premix Ex Taq TM (Takara, Kusatsu, Shiga, Japan) according to the manufacturer's protocol. Data were then processed using the 2^^−ΔΔCT^ method. The sequences of genes including *Gapdh*, *Il‐6*, and *Il‐6r* their primer pairs were listed as follows:

*Il‐6‐*F: CTGCAAGAGACTTCCATCCAG

*Il‐6‐*R: AGTGGTATAGACAGGTCTGTTGG

*Il‐6r*‐F: CCTGAGACTCAAGCAGAAATGG

*Il‐6r*‐R: AGAAGGAAGGTCGGCTTCAGT

*Gapdh*‐F: AGGTCGGTGTGAACGGATTTG

*Gapdh*‐R: TGTAGACCATGTAGTTGAGGTCA

### RNA Sequencing

According to the above methods, hippocampal astrocytes were isolated and total RNA was extracted by Trizol method. The synthesized cDNA library was constructed according to the Illumina library construction protocol, and then the library was sequenced using the Illumina NovaSeq 6000 sequencer (read length 2×150 bp). Fastp software (https://github.com/OpenGene/fastp) was utilized for cutting and quality control of the raw sequencing data. Then HISAT2 (http://ccb.jhu.edu/software/hisat2/index.shtml) and StringTie (https://ccb.jhu.edu/software/stringtie/) were used for evaluating the quality of transcriptome sequencing results, including sequencing saturation, gene coverage, distribution of Reads in different regions of the reference genome, and distribution analysis of Reads in different chromosomes. Then RSEM software (http://deweylab.github.io/RSEM/) was utilized to calculate the expression of gene transcription or the quantity, the unit of the quantitative results is TPM/FPKM, which homogenization process made the total expression in different samples consistent. DESeq2 (http://bioconductor.org/packages/stats/bioc/DESeq2/) is based on negative binomial distribution model and it was used to read count data to screen differential expressed genes. The default screening criteria for significantly DEGs were: FDR < 0.05 & |log2FC| ≥1, when a gene satisfy these two conditions, this gene was recognized as the DEGs.

### Immunofluorescence and Images Analysis

Mice were anesthetized with 2% pentobarbital sodium and were perfused transcardially with 0.9% saline before brain collection. Then the whole brain was placed in 4% paraformaldehyde overnight at 4 °C. After dehydration with a gradient of 30% sucrose‐PBS solution, 35 µm brain slices were prepared through Cryostat Microtome (Leica, CM1850, Germany). Briefly, brain sections were blocked with 10% goat serum and incubated for 2 h at room temperature (RT) and then incubated with primary antibodies Iba1 (Rabbit Polyclonal, 1:500, FUJIFILM Wako Pure Chemical Corporation, 019–19741, Japan), GFAP (Mouse mAb, 1:500, Abcam, ab279289, USA), IL‐6R (Rabbit, 1:500, Abcam, ab300581, USA) overnight at 4 °C. Then, the brain sections were incubated with Alexa Fluor 488 (1:500, Invitrogen, A‐11034, USA) or Alexa Fluor 594 (1:500, Invitrogen, R37115 and A‐21207, USA) secondary antibody for 2 h at RT, and then washed 3 times for 15 min with PBS. Nucleus was stained with DAPI (Southernbiotech, 0100–20, DAPI Fluoromount‐G, Birmingham, AL, USA). Images were obtained and analyzed using Olympus FV1000 confocal microscope (Olympus, FV1000, Japan).

### 3D Reconstruction

The 30 µm coronal slices were stained with primary antibodies GFAP (Mouse mAb, 1:500, Abcam, ab279289, USA) for 24 h, followed by Alexa Fluor 594‐conjugated secondary antibody (1:500, Invitrogen, R37115 and A‐21207, USA) staining and nuclei were counterstained with DAPI. Imaging was performed on a NIKON microscope (AIR‐MP). Z stacking was performed with 0.12 µm per step in the Z direction for 60 slices. Analyzed was used IMARIS 9.6.2 software (Bitplane) and the IMARIS function “‘Filaments”’ was used to quantify process length and number of branches, and the function “Surface” quantified the surface area of cells. The Sholl analysis was also used by “Filaments”. Two images randomly picked from per mouse were reconstructed, which each had at least 10 cells, for 6 mice in each group. The mean result was used for morphological analysis.

### Terminal Deoxynucleotidyl Transferase‐Mediated dUTP‐Biotin Nick end Labeling (TUNEL) Assay

TUNEL staining was performed with Tunel kit (Servicebio, China). The paraffin sections were subjected to a dewaxing process, followed by the execution of antigen retrieval. Subsequently, Buffer reagent was administered to the surface to completely cover the sections, and incubation was carried out at RT for 10 min. According to the number and the size of the slices, an appropriate amount of reagent 1 (TdT) and reagent 2 (dUTP) were taken from the Tunel kit, and they were mixed with Buffer reagent at a ratio of 1:5:50, and incubated in 37 °C for 2h. Then brain sections were blocked with 10% goat serum and incubated for 2 h at RT and then incubated with primary antibodies GFAP (Mouse mAb, 1:500, Abcam, ab279289, USA) overnight at 4 °C. Then, the brain sections were incubated with Alexa Fluor 594 (1:500, Invitrogen, R37115 and A‐21207, USA) secondary antibody for 2 h at RT. Nucleus was stained with DAPI. Images were observed with an Olympus FV1000 fluorescence microscope.

### Western Blotting (WB)

The hippocampus and cultured astrocytes were collected for protein sample. After gel electrophoresis, PVDF membranes were blocked with 5% nonfat milk at RT for 2h and then incubated overnight at 4 °C with primary antibody. After washing with TBST, PVDF membranes were incubated for 2 h at RT with a secondary antibody. Immunoactivity was detected by ECL reagent (Millipore, USA). The proteins were probed with respective primary antibodies, including Bax (1:1000, abcam, ab32503, USA), Bcl‐2 (1:1000, Proteintech, 12789‐1‐AP, USA), Caspase‐3 (1:500, Proteintech, 19677‐1‐AP, USA), p62 (1:1000, Proteintech, 18420‐1‐AP, USA), and LC3(1:1000, Novus, NB100‐2220, USA). The membranes were probed with GAPDH antibody (1:5000, Proteintech, HRP‐60004, USA) and β‐actin (1:5000, Proteintech, 66009‐1‐Ig, USA) as internal control. ImageJ was used for following analysis of immunoblots.

### Transmission Electron Microscopic (TEM) Analysis

Mice were perfused with saline, and a small portion (≈1 mm^3^) of the hippocampus was sectioned and incubated in glutaraldehyde for 2 h at 4 °C. Specimens were postfixed in 1% osmium tetroxide, stained in aqueous uranyl acetate, and then dehydrated and embedded in epoxy resin. Ultrathin sections were stained using lead citrate and examined with a transmission electron microscope (JEM‐1010, Tokyo, Japan). When examined under the electron microscope, astrocytes exhibited sparse cytoplasm, well‐defined nuclei with a peripheral ring of dense chromatin, a substantial presence of staggered fibrils within the cytoplasm, a paucity of free ribonucleoproteins and rough endoplasmic reticulum, and an abundance of glycogen granules.

### Virus Injection and Cannula Implanted

The AAV2/5‐CBG‐DIO‐EGFP‐miR30shRNA(Il6ra)‐WPRE virus (AAV2/5‐DIO‐IL6R‐shRNA) was engineered to downregulate IL‐6 receptor (IL‐6R) expression in hippocampal astrocytes, with AAV2/5‐CBG‐DIO‐EGFP‐miR30shRNA(Scarmble)‐WPRE (AAV2/5‐DIO‐EGFP) serving as a control virus. Additionally, AAV‐MG1.2‐SFFV‐DIO‐EGFP‐5′miR‐30a‐shRNA(mIL‐6)‐3′miR‐30a (AAV‐MG1.2‐DIO‐IL6‐shRNA) was developed to downregulate IL‐6 expression in hippocampal microglia, with AAV‐MG1.2‐SFFV‐DIO‐EGFP‐5′miR‐30a‐shRNA(Scarmble)‐3′miR‐30a (AAV‐MG1.2‐DIO‐EGFP) as the control virus.

For in vivo viral injection, viral vectors were bilaterally targeted to the hippocampus (Bregma = 2.0 mm; L = ± 1.5 mm; V = 1.8 mm). Animals received intracranial viral injections while under 2% pentobarbital anesthesia using a stereotactic apparatus (RWD Life Science, Shenzhen, China). Mice were secured using ear bars and a head holder. A microsyringe pump controller was used to inject viral vectors (5 min for 0.25 µL into each hippocampus). After injection, the syringe was held in place for 10 min to avoid backflow. Animals were sutured and placed on a heating pad for recovery.

### Flow Cytometry

Apoptosis flow assays were performed using an Annexin V‐FITC/PI apoptosis kit (BD Pharmingen). At the end of the manipulation, the astrocyte supernatant was discarded and the cells were washed for 3 times with HBSS (Corning) without Ca^2+^ and Mg^2+^. The cells were then digested with Accutase solution (Cat. No. A6964, MCE) at 37 °C for 30 min, and the cells were gently collected. Then cells were resuspended in 1 × binding buffer at a concentration of 1.5 × 10^5^/mL. For detection, 5 µL FITC and 5 µL PI were added to each tube and stained at RT for 15 min before testing. Subsequent detection was performed with FlowJo.

### Enzyme‐Linked Immunosorbent Assays (ELISA)

The microglia supernatant was centrifuged at 300 g for 10 min to remove the precipitate and collect the supernatant. And the hippocampus was homogenized in lysis buffer containing protease (Beyotime, China) and phosphatase inhibitors (MCE, USA) at 4 °C, then the hippocampal lysate was subjected to centrifugation at 12000 × g for 15 min to acquire supernatant. ELISA was performed following the manufacturer's instructions (Multisciences Biotech Co., Ltd., China) to quantify the IL‐1β, IL‐4, IL‐6, TNF‐α, and IL‐10 content in the supernatant. OD values were detected within 30 min at a wavelength of 450 nm, with reference wavelengths of 570 nm or 630 nm.

### Statistical Analysis

The QQ‐plots were used to verify the assumptions before t test, ANOVA and descriptive statistics. Statistical comparisons between two groups were conducted using Student's *t*‐tests. One‐way and Two‐way analysis of variance (ANOVA) and Bonferroni post hoc analyses were used in analyses with multiple experimental groups. Data are shown as individual values or expressed as the mean ± SEM, and significance levels are indicated as *p < 0.05, **p < 0.01, ***p < 0.001 and not significant (n.s.). Analyses and graphing were conducted in GraphPad Prism 9.4 (GraphPad Software).

## Conflict of Interest

The authors declare no conflict of interest.

## Author Contributions

S.S. and L.L. contributed equally to this work. Conceptualization was performed by J. Yu. Methodology was performed by S. Shen, L. Liang, and T. Shi. Formal analysis was performed by S. Shen, L. Liang, and S. Yin. Investigation was performed by S. Shen, L. Liang, Z. Shen, J. Zhang, W. Li, and W. Mi. Writing —original draft was performed by S. Shen and L. Liang. Writing—review & editing was performed by S. Shen, L. Liang, Y. Zhang, and J. Yu. Resources were taken care of by Y. Wang, Y. Zhang, and J. Yu. Funding acquisition was performed by Y. Wang, Y. Zhang, and J. Yu. Supervision was performed by Y. Zhang and J. Yu.

## Supporting information



Supporting Information

## Data Availability

The data that support the findings of this study are available from the corresponding author upon reasonable request.
