# Supplementary material for: Microglia‐Derived Interleukin‐6 Triggers Astrocyte Apoptosis in the Hippocampus and Mediates Depression‐Like Behavior
Source: Adv Sci (Weinh). 2025 Jan 30;12(11):2412556. doi: 10.1002/advs.202412556 (PMC11923973; doi:10.1002/advs.202412556)
Supplement: Supplementary file 1 — Supporting Information [file ADVS-12-2412556-s001.docx]

Supporting Information

Microglia-derived Interleukin-6 Triggers Astrocyte Apoptosis in the Hippocampus and Mediates Depression-like Behavior

Shi-Yu Shen, Ling-Feng Liang, Tian-Le Shi, Zu-Qi Shen, Shu-Yuan Yin, Jia-Rui Zhang, Wei Li, Wen-Li Mi, Yan-Qing Wang, Yu-Qiu Zhang*, Jin Yu*

**
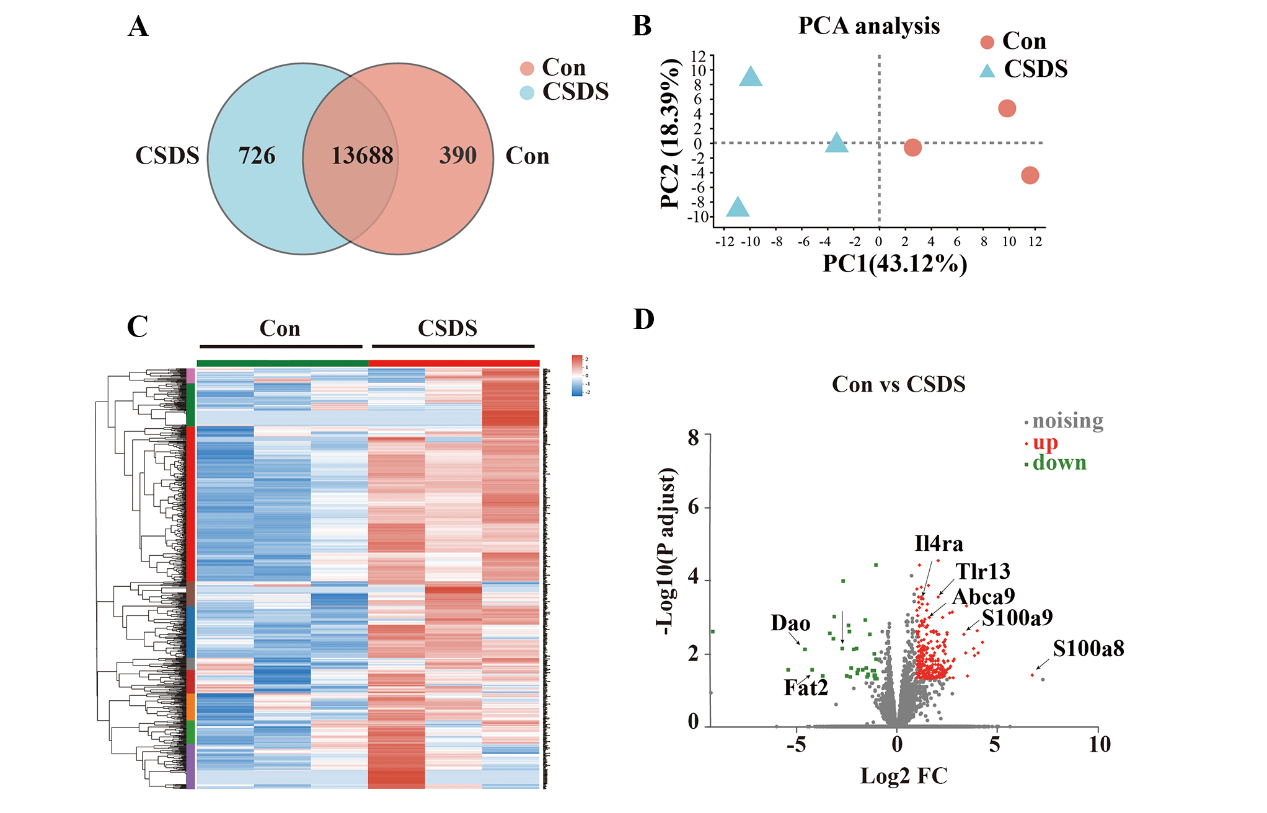
**

**Figure S1 Transcriptome of hippocampal astrocytes changes after CSDS.**

(A) Venn analysis between control and CSDS mice.

(B) Principal component analysis (PCA) between samples for control and CSDS mice.

(C) Heat map of DEGs after transcriptome RNA sequencing of hippocampal astrocytes of control and CSDS mice, and the expression of DEGs is represented by normalized z-score values (up-regulated expression is red, down-regulated expression is blue).

(D) Volcano plot of DEGs between control and CSDS mice.


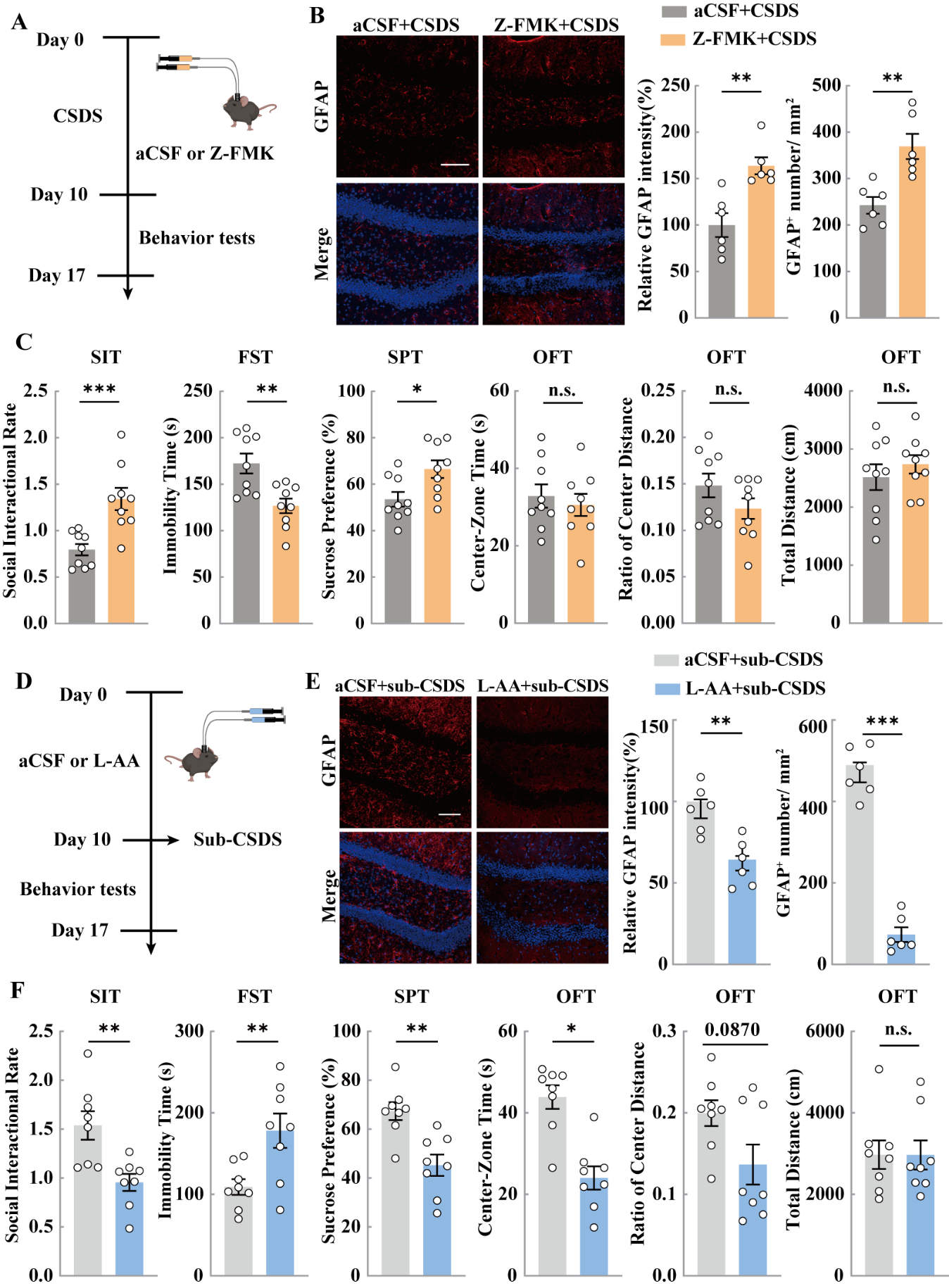


**Figure S2.** **Manipulation of astrocytes affects anxiety- and depression-like behaviors.**

1. Experimental timeline of Z-DEVD-FMK (Caspase-3 inhibitor, Z-FMK) treatment and behavioral tests.
2. Representative images of GFAP (red) staining in the hippocampus of CSDS mice treated with artificial cerebrospinal fluid (aCSF) or Z-FMK through an infusion cannula. Quantitative immunostaining analysis and number of astrocytes positively labeled for GFAP are shown right. Scale bars = 50 μm. Unpaired *t* test. n = 6.
3. Performance of CSDS mice treated with aCSF or Z-FMK in SIT, FST, SPT, and OFT. Unpaired *t* test. n = 9.
4. Experimental timeline of L-α-aminoadipate (L-AA) treatment and behavioral tests.
5. Representative images of GFAP (red) staining in the hippocampus of sub-CSDS mice treated with aCSF or L-AA through an infusion cannula. Immunostaining quantitative analyses and number of cells positively labeled for GFAP are shown right. Scale bars = 100 μm. Unpaired *t* test. n = 6.
6. Performance of sub-CSDS mice treated with aCSF or L-AA in SIT, FST, SPT, and OFT. Unpaired *t* test. n = 8. All data are shown as mean ± S.E. M, * *p* < 0.05, ** *p* < 0.01, *** *p* < 0.001.


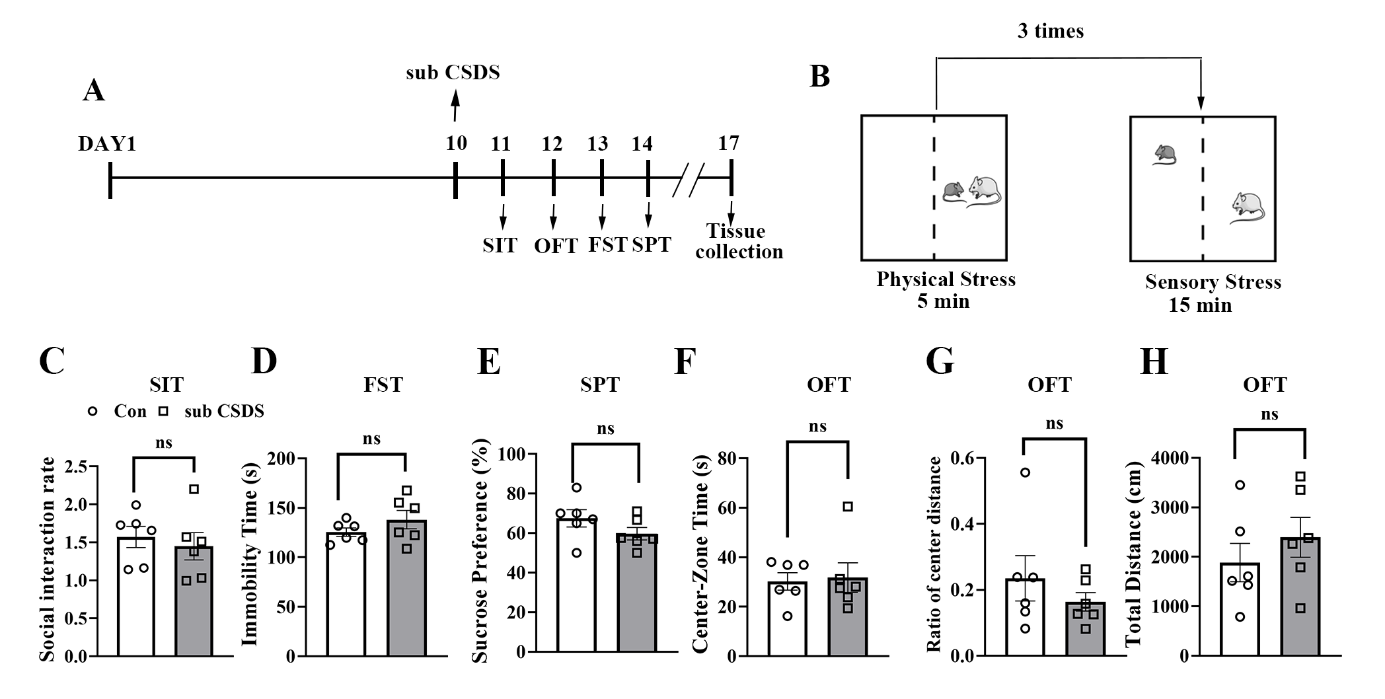


**Figure S3. The sub CSDS model did not induce anxiety- and depression-like behaviors.**

(A) Preparation of sub CSDS model and subsequent testing process of behavioral phenotype.

(B) Schematic of sub CSDS procedure.

(C)Social rate (SIR) in the Social Interaction test (SIT).

(D)Immobility time in the forced swimming test (FST).

(E) Degree of sucrose preference in the Sucrose Water Preference test (SPT).

(F-H) Central zone movement time (F), central zone movement ratio (G), and total distance traveled (H) in the open field test (OFT).


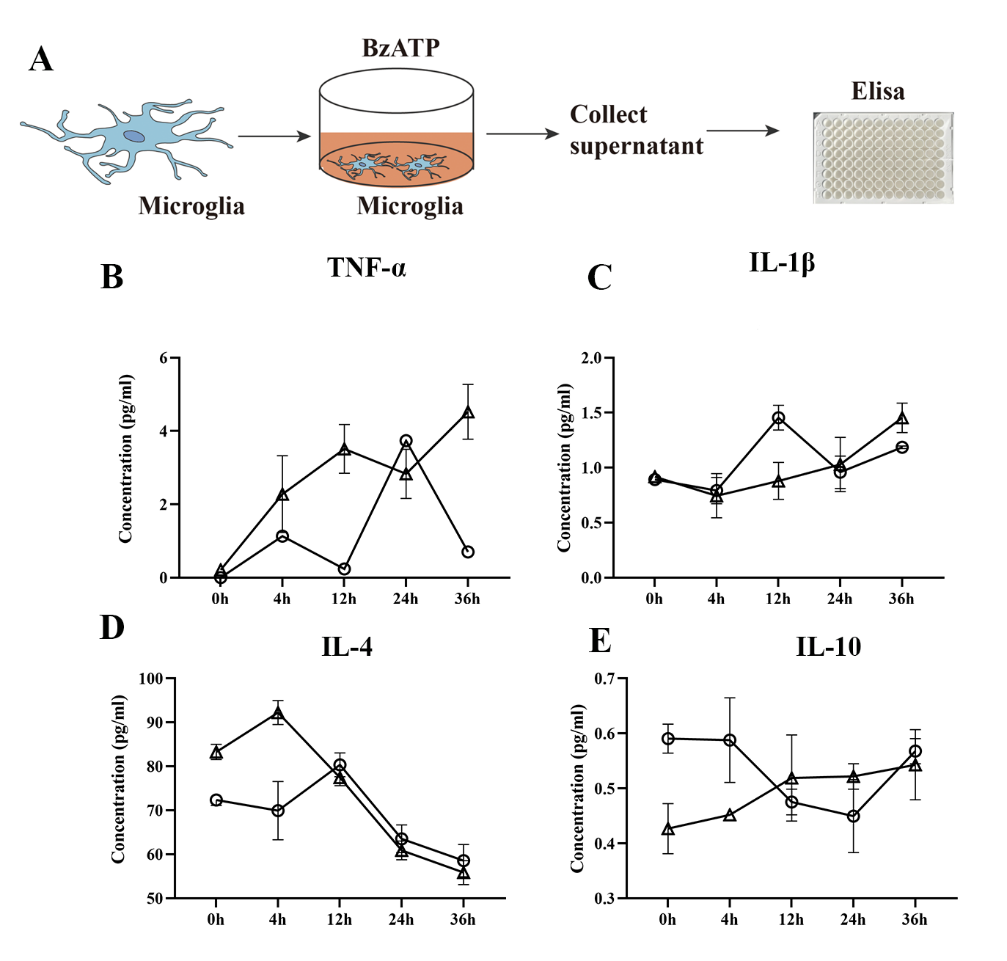


**Figure S4. Detection of cytokines released by microglia after BzATP treatment.**

1. Schematic diagram of detecting inflammatory cytokines released from microglia by Elisa.

(B-D) Cytokines (IL-4, IL-10, TNF-α, IL-1β) secretion by microglia under PBS and BzATP treatment. Two-way ANOVA.


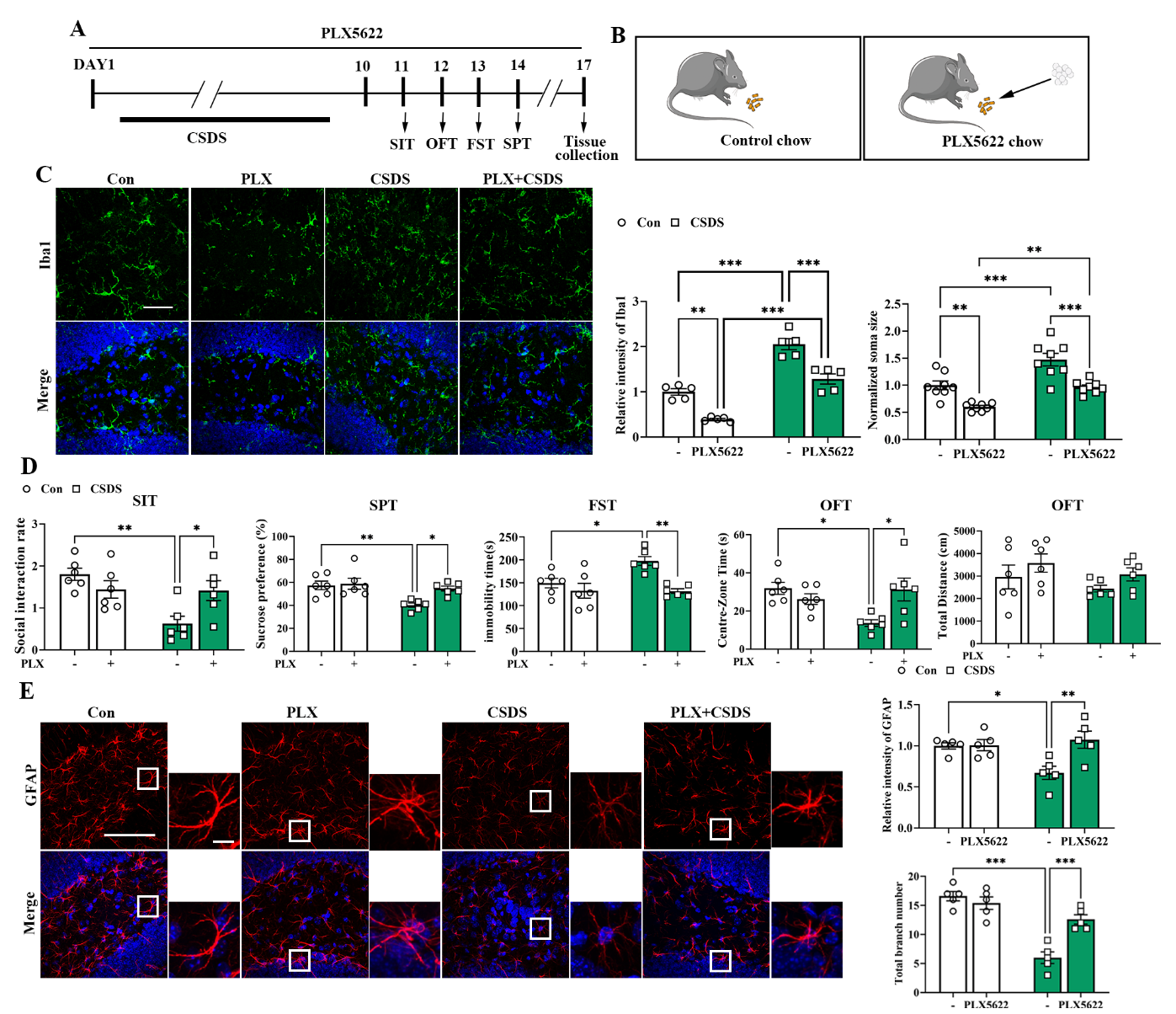


**Figure S5. Microglial depletion alleviates anxiety- and depression-like behaviors, and ameliorates astrocytic loss.**

(A) Experimental timeline of PLX5622 treatment, CSDS protocol and behavioral tests.

(B) Schematic of PLX5622 diet and the control diet.

(C) Representative images of Iba1 (green) staining in the hippocampal DG of Con and CSDS mice fed with PLX5622 or control chow. Quantitative immunostaining analysis (n = 5) and normalized soma size of microglia (n = 8) are shown right. Scale bars = 50 μm. Two-way ANOVA.

(D) Performance of Con and CSDS mice fed with PLX5622 or control chow in SIT, SPT, FST and OFT. Two-way ANOVA. n = 6.

(E) Representative images of GFAP (red) staining in the hippocampal DG of Con and CSDS mice fed with PLX5622 or control chow. Quantitative immunostaining analysis are shown (right). Scale bars (overview) = 40 μm and scale bars (magnified) = 10 μm. Two-way ANOVA. n = 5. All data are shown as mean ± S.E.M, * p < 0.05, ** p < 0.01, *** p < 0.001.

**
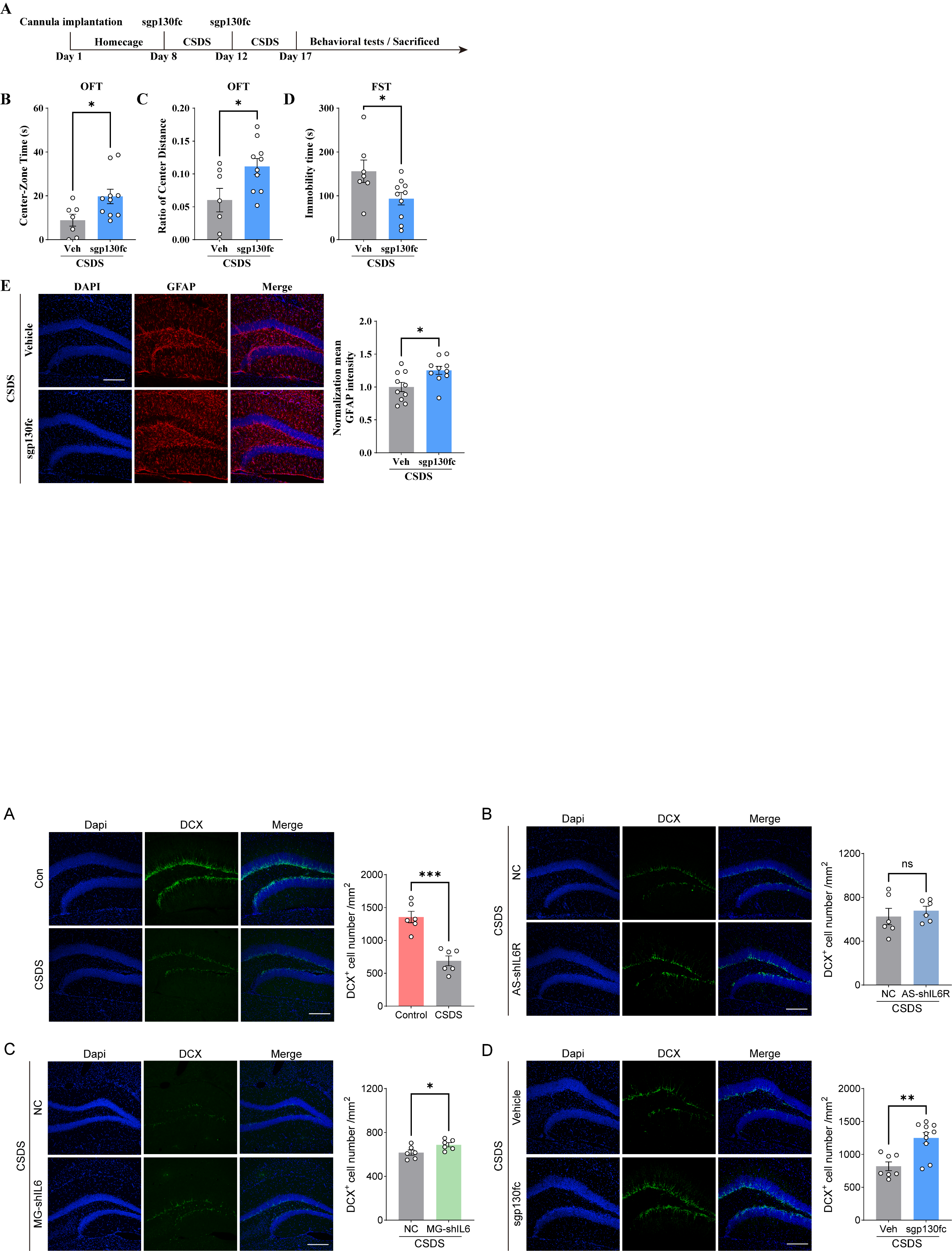
**

**Figure S6. Sgp130 treatment alleviates anxiety- and depression-like behaviors, and ameliorates astrocytic loss.**

1. Experimental timeline of sgp130fc treatment, CSDS protocol and behavioral tests.
2. Center-Zone time in open field test (OFT). Unpaired *t* test. n_Veh_=7, n_sgp130fc_=10.
3. Ratio of Center Distance in open field test (OFT). Unpaired *t* test. n_Veh_=7, n_sgp130fc_=10.
4. Immobility time in forced swimming test (FST). Unpaired *t* test. n_Veh_=7, n_sgp130fc_=10.
5. Representative images of GFAP immunostaining (red) in the hippocampus of Veh or sgp130fc mice (left). Quantitative immunostaining analysis of Veh and sgp130fc mice (right). Unpaired *t* test. n_Veh_=7 slices from 7 mice, n_sgp130fc_=10 slices from 10 mice. Scale bars = 200 μm. All data are shown as mean ± S.E.M, * p < 0.05, ** p < 0.01, *** p < 0.001.
